# High-resolution body-surface electrocardiograph system and survey of possible applications

**DOI:** 10.1186/s40064-015-1325-8

**Published:** 2015-09-21

**Authors:** Yasushi Toyosu, Shigeru Inui, Zhong Wang, Masatake Akutagawa, Shinsuke Konaka, Yohsuke Kinouchi

**Affiliations:** Department of Electrical and Electronic Engineering, Faculty of Engineering, Graduate School of Advanced Technology and Science, The University of Tokushima, 2-1 Minamijosanjimacho, Tokushima, 770-8506 Japan; Yukari Tech Co., Ltd., 1-5-9, Kanazawa, Tokushima 770-0871 Japan; Center for Statistical Genetics Cancer Institute, Pennsylvania State University, 500 University Driver, P.O. Box 850, Hershey, PA 17033-0850 USA; Institute of Technology and Science, University of Tokushima, 2-1 Minamijosanjimacho, Tokushima, 770-8506 Japan

**Keywords:** Electrocardiograph, Body-surface potential, Spring-loaded electrodes

## Abstract

A body-surface electrocardiograph system employs unique spring-loaded metal-rod electrodes encased in metal housings to minimize set-up time and noise. 124 electrodes spaced at 35 mm intervals acquire body-surface potential with a 10 kHz sampling rate to capture and image (time sequentially) electrical activity of the heart not observable with standard 12-lead electrocardiography. Possible applications surveyed include assessing cardiopulmonary facility, examining age-related effects, and quantifying warning signs for myocardial infarction.

## Background

The standard 12-lead electrocardiograph (ECG) has been widely accepted for general understanding of cardiac activity (Jacobson and Webster 1997; Waller 1889). The 12-lead ECG attaches adhesive electrodes to the body to capture heart cell electrical behavior as one spatially-composite voltage that varies as a function of time. However, the amount of information acquired by the 12-lead ECG is limited, and its measurement accuracy, reproducibility, and error-dependence on equipment manufacturer are issues (Lepeschkin 1974; Sridharan and Horan 1988).

As early as the 1980s, a number of researchers sought more comprehensive understanding of the heart’s electrical behavior by radically increasing the number of electrodes, and their efforts resulted in body-surface electrocardiography (Harumi et al. 1993; Yasui 1994). Body-surface electrocardiography places numerous electrodes on the surface of the body near the heart, acquires voltage synchronized to heart-beat at each electrode, and produces body-surface potential maps in two spatial dimensions. By collecting data at more locations over a wider area and not limiting measurement results to a single time-varying potential at each of the 12-leads (as in standard 12-lead ECG), body-surface electrocardiography has verified that the heart’s electrical behavior is not single-faceted but rather is a composite of multiple phenomena. It was anticipated that body-surface potential mapping (BSPM) could acquire information that was formerly difficult to measure, and in particular, diagnosis of cardiovascular disease was targeted as a possible application (Taccardi et al. 1998). However, early body-surface electrocardiograph systems adhesively-attached many electrodes to the subject’s body, which required significant preparation time and made it difficult to achieve stable electrical contact at all electrodes. In addition, electrode attachment location was difficult to duplicate for re-measurement, measurement sampling rate was low compared to the rapidly varying cardiac potential, and data presentation was in the form of potential maps that were printed-out one at a time. These types of unresolved problems have impeded full acceptance of body-surface electrocardiography extending to the present. The 12-lead ECG is still the primary tool used in medical practice, and BSPM has yet to supplant the 12-lead ECG (Taccardi et al. 1998).

To resolve the problems cited above, the authors (and associates) developed a high-resolution body-surface electrocardiograph system (Inui et al. 2010; Inui et al. 2007). Primary features of the system are described below. The system was used to acquire body-surface electrocardiograph data for subjects with cardiovascular disorders as well as healthy subjects. Although 140 subjects including 60 healthy subjects and 80 subjects suffering cardiovascular disorders cooperated with this study, the number of subjects suffering a single type of ailment was limited. At this stage, statistical analysis of the acquired data is incomplete. However, difference in body-surface potential maps for each health condition was clear. A survey of five different types of cardiac-function-related measurements indicated that the system may be capable of capturing (heart-related) physiologic phenomena undetectable or not clearly observable with the 12-lead ECG.

## Methods

### Body-surface electrocardiograph system

**Fig. 1:**
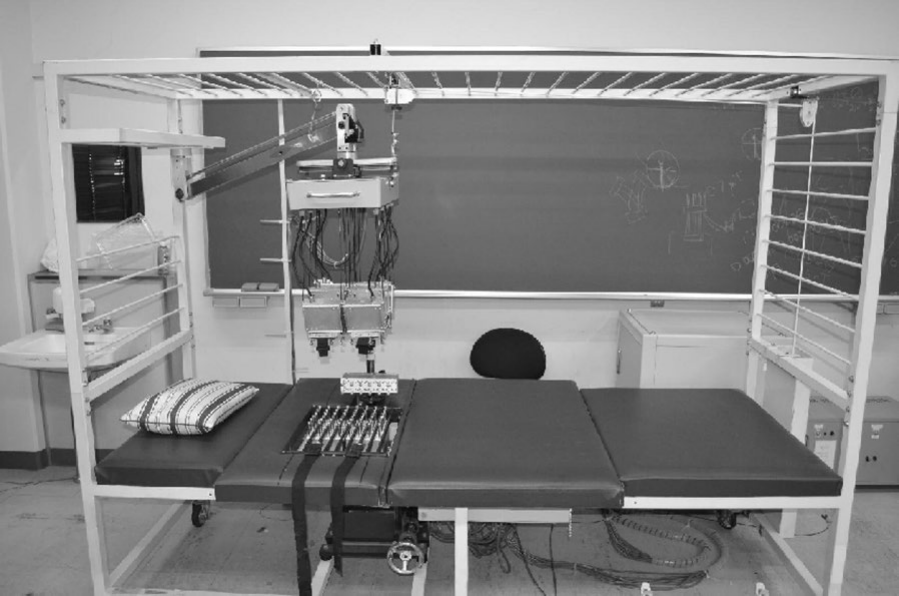
Photograph showing the system measurement platform where the subject reclines for contact with the electrode array (slightly *left of center*)

**Fig. 2:**
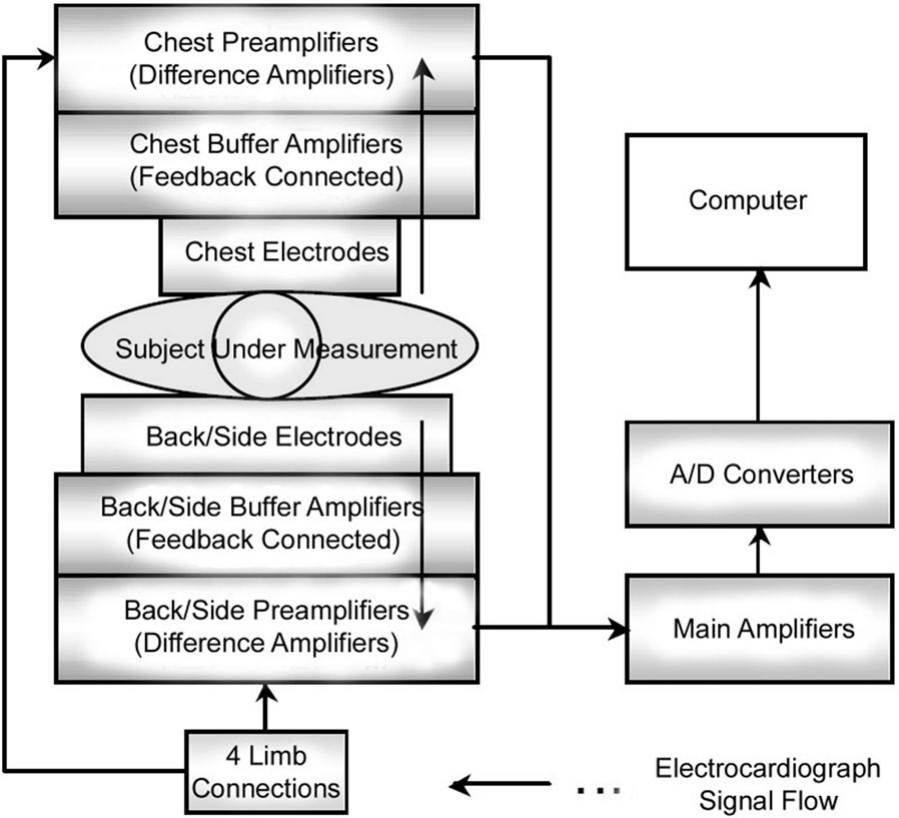
Simplified schematic showing overall system organization and electrocardiograph signal flow

**Fig. 3:**
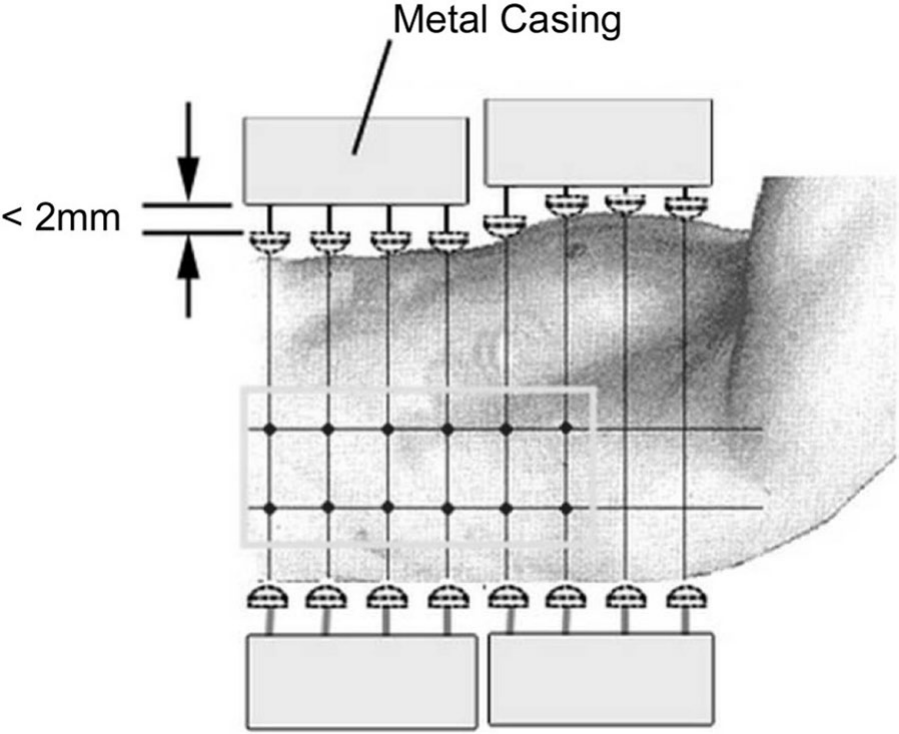
Side view schematic drawing showing electrodes in contact with the body; side electrodes are shown as *blue dots* inside a *yellow rectangular* housing

Although numerous body-surface electrocardiograph systems of various types have been reported, standard specifications have yet to be established and development is still at a prototype level. The following outlines characteristics and features of the system developed by the authors with comparison to the conventional 12-lead ECG and earlier body-surface electrocardiograph systems. Figure 1 is a photograph of the system, Fig. 2 is a system schematic showing signal flow, and Fig. 3 is a schematic drawing showing electrode contact to the body for measurement.

#### Features

##### High temporal resolution

Measurements are made with a 10 kHz sampling rate, which is three times that of the 12-lead ECG and twenty times that of earlier body-surface electrocardiograph systems.

##### High spatial resolution

**Fig. 4:**
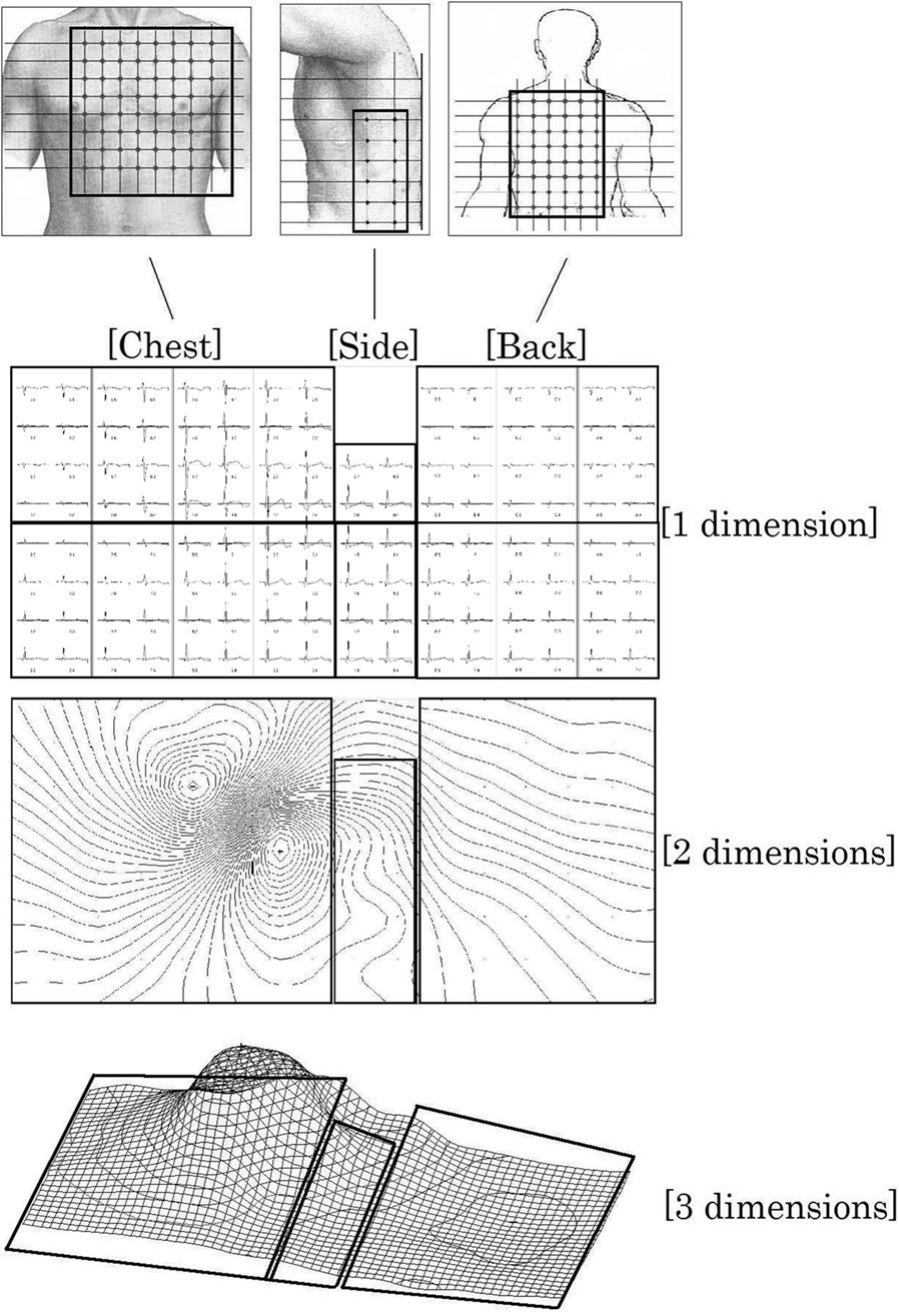
Sketch of the body and three plots showing the relation between electrode position on the body, waveforms at each electrode, the 2D body surface map (BSM), and the 3D BSM

A total of 124 electrodes are disposed on the chest, underarm, and back. This is ~20× the six chest points of the conventional 12-lead ECG and ~1.5× the 84 points of earlier body-surface electrocardiograph systems. As shown in Fig. 4, the system measures body-surface potential with an 8 × 8 = 64 electrode array on the chest, a 6 × 2 = 12 electrode array on the side (underarm), and an 8 × 6 = 48 electrode array on the back (Inui et al. 2008; Inui et al. 2007).

##### Electrode housing improves noise immunity and conforms to the body

As shown in Fig. 3, noise reduction is improved by housing electrodes in box-shaped metal casings. The distance from the tip of each electrode to its dedicated amplifier is kept short at ~ 10 cm, amplifiers are mounted inside the metal electrode casings, and those metal casings cover the entire region near the heart to act as noise shielding. Further, since electrodes are pushed into the interior of the housings with body contact, metal casings are drawn closer to the body-surface to increase noise shielding during measurement. Effective shielding has been demonstrated up to 71.92 μT, which is in the vicinity of the 60 Hz, 83.3 μT guideline value of the International Commission on Non-Ionizing Radiation Protection (ICNIRP). Further, when measurements were made intentionally exposing the measurement platform to high amplitude electromagnetic radiation (noise), some noise effects were observable but large differences in measurement values between the high-noise and normal environment were not observed. Accordingly, noise shielding is sufficient to measure body-surface potential under normal (low radiation) conditions. In addition, electrode casings are joined in a ball-joint fashion allowing independent movement and rotation. Each electrode casing can incline independently to conform to the contour of the body making it easy to establish reliable contact with accurate electrode positioning.

##### Spring-loaded electrodes make stable electrode-to-body contact

As shown in Fig. 3, electrodes are pressed against the body and retract in a spring-loaded manner into the electrode housings. Polytetrafluoroethylene, resin (PTFE, Teflon^®^) electrode guides are built into the electrode housings to reduce friction with the spring-loaded electrode rods, which move in and out to conform to the body-surface. By applying pressure with metal springs, approximately the same electrode pressure can be achieved on the chest, side, and back. When a subject breathes during measurement, the contour of the body is physically altered. Since each spring-loaded electrode applies pressure independently, electrode lateral position shift is minimized and spring-loaded extension and retraction during breathing allows each electrode to follow body movement and maintain a consistent state of contact. Each electrode rod has a copper-zinc alloy hemispherical head that increases the contact area to reliably detect electric potential induced on the body-surface. Electrode diameter is 1.0 cm with a 35 mm spacing between center-points of adjacent electrodes. With this electrode design, manual thus time-consuming adhesive-attachment of individual electrodes in conventional body-surface electrocardiograph system is avoided, and since the electrodes are reusable, there is no waste of resources and no additional recurring costs.

##### No band-eliminating (notch) filtering (no hidden data)

12-lead ECG as well as body-surface electrocardiograph systems currently in use employ a band-eliminating filter to eliminate noise at commercial alternating current (AC) frequencies. Since there are no international standards for this type of filter, each electrocardiograph system has its own manufacturer-specific filter. Our studies indicate that the time-varying electro-potential distributions are altered by filtering. Electrocardiograph signals are in a frequency range on the order of 0.05 to 100 Hz and filtering in that range distorts waveforms and prevents accurate signal capture. Accordingly, the system does not include a band-eliminating filter.

##### Three-dimensional (3D) graphical representation promotes insight

With electrodes disposed on the chest, left side, and back as shown in Fig. 4, single spatial dimension potential waveforms (similar to 12-lead ECG waveforms) are obtained as shown by the upper chart of Fig. 4, and combination of those waveforms (at a given time) can produce the two-dimensional (2D) equipotential map shown in the center plot of Fig. 4. Further, by representing potential as a third vertical dimension, the 2D map can be illustrated three-dimensionally as shown in the lower representation of Fig. 4, and consecutive (time) sequencing of those 3D plots presents an animated record of the heart’s electrical activity. This has the merit that body-surface potential distribution attributes, which change rapidly in a complex manner, can be quickly grasped in an intuitive manner. Graphic-related descriptions below are based on animated 3D graphical representation. The 2D base of the 3D plots corresponds to spatial coordinates on the surface of the subject’s body, and potentials at three equidistant points between adjacent electrode locations (35 mm) are computed by interpolation. Accordingly, the 3D time-varying grid has an 8.75 mm pitch between grid-points.

#### Specifications

Important specifications of the high-resolution system are as follows:

- Number of channels: 124.
- Maximum input voltage (V_in_): 5 mV and 10 mV (dual range).
- Resolution: 10 bit.
- Least-significant-bit (LSB) value: 500 μV and 1 mV (depending on V_in_).
- Sampling period: 0.1 ms.

In the same manner as in the 12-lead ECG, potential at electrodes attached to both arms and the left leg are averaged to establish the Wilson central terminal used as the ground potential to determine voltage at each electrode. Voltage signals induced on the body-surface are acquired at each electrode, amplified (with total gain = 1000) through a buffer amplifier, pre-amplifier, and main amplifier, converted to digital values in an analog-to-digital (A/D) converter, and read into a computer for 2D and 3D display as well as other data processing.

## Discussion

### Survey of possible applications

To evaluate how features and characteristics of the high-resolution body-surface electrocardiograph system (also subsequently referred to as the present system) could be applied advantageously, and to survey possible uses of the system such as in a clinical environment, measurements considered difficult to detect with the standard 12-lead ECG were attempted. The system was used to acquire data from 80 subjects in an age range from 30 to 80 suffering cardiovascular disorder and 60 healthy subjects in an age range from 10 to 70 (patients of the Tokushima University Hospital, Tokushima University students, and other associates). 29 % of the subjects were female, and 71 % male. Measurements were repeated three times over a 10 min period with the subject resting quietly face-up on a bed built into the system. Subject acceptance of electrode contact was generally good with some comments concerning coldness of the metal electrode tips. Measurements were made with approval of the Tokushima University Medical and Dental School Hospital Clinical Research Ethics Committee and informed consent of the subjects. The following assessments were made and considered as candidates for useful application of the present system:

1. appraisal of cardiopulmonary faculty;
2. evaluation of changes in heart function with age; and
3. possible detection of myocardial infarction warning signs.

The number of different conditions was limited with an eye on the possibility for future useful clinical application of the present system.

#### Differences between normal healthy subjects and athletes

**Fig. 5:**
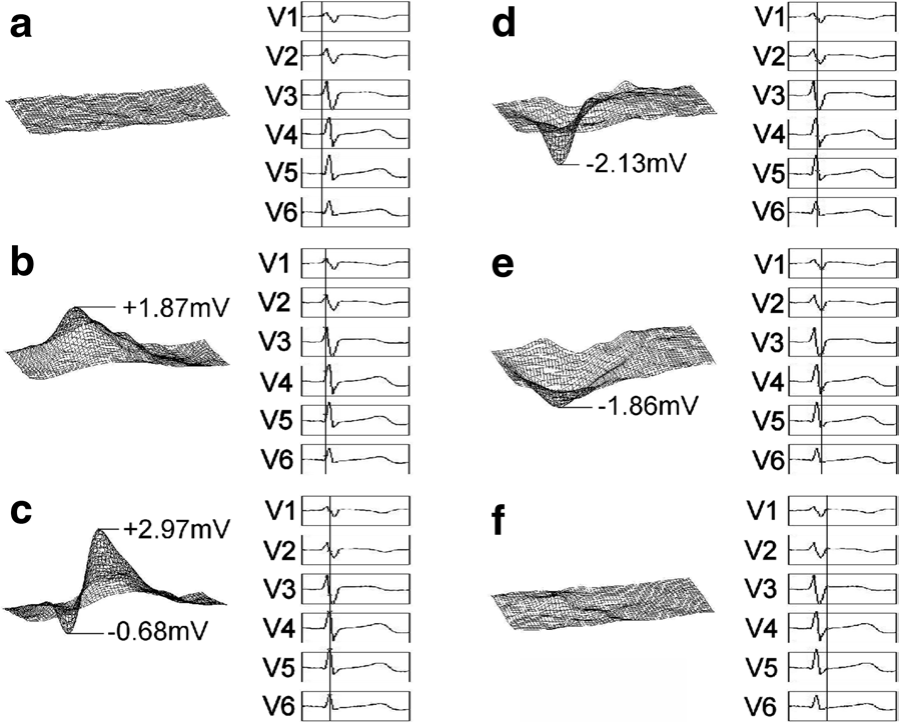
Body-surface potential for a healthy 20 year old male; equivalent V1–V6 waveforms are shown for reference with the vertical time-line indicating capture time of the 3D dynamic representation (**a**–**f**)

**Fig. 6:**
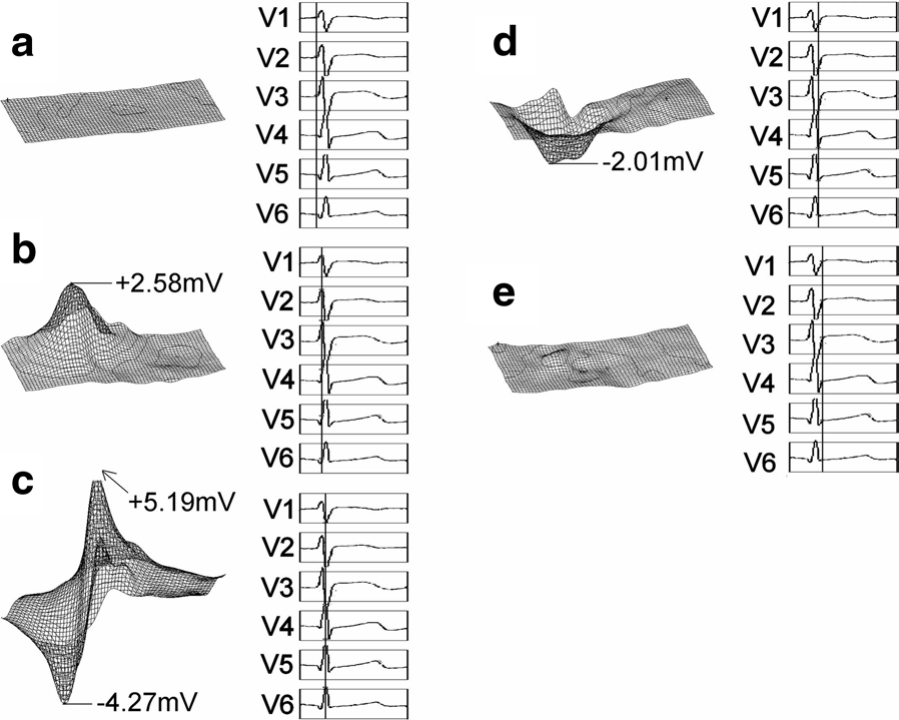
Body-surface potential for a well-conditioned 20 year old male swimmer showing 3D dynamic representation (**a**–**e**); **c** shows simultaneous positive and negative peaking observed only for well-conditioned athletes

First, body-surface electrocardiograph data was collected for a healthy subject as a benchmark for appraising potential distribution maps. Body-surface potential distribution for a particularly well-conditioned athlete (competitive swimmer) was compared with the healthy subject’s data. Figure 5 shows body-surface potential for the healthy subject and Fig. 6 shows that for the well-conditioned athlete. Both subjects were 20 year old males. Waveforms at the standard 12-lead ECG V1–V6 chest-lead positions are also shown along with each potential distribution. V1–V6 waveforms are extracted from measurement data at corresponding body-surface electrocardiograph system grid-points as shown in the 2D map of Fig. 4, and time-points for corresponding 3D potential distributions are indicated by vertical lines through the V1–V6 waveforms.

Figure 5b shows right ventricle maximum positive potential, and the subsequent plot of Fig. 5c shows the potential distribution when the left ventricle attains maximum positive potential (R peak). Taking advantage of the 10 kHz sampling rate, our measurements have shown that delay between the right ventricle positive maximum and the left ventricle positive maximum is on the order of 10 ms. This delay time has not been noted in previous 12-lead ECG measurements. Next, Fig. 5d shows the potential distribution at the point of maximum negative potential, and Fig. 5e corresponds to the minimum value of the V5 chest-lead waveform.

In contrast, the potential distribution for the competitive swimmer of Fig. 6 shows different characteristics. Figure 6b shows right ventricle maximum positive potential, Fig. 6c shows left ventricle maximum positive potential (R peak), and Fig. 6d shows the V5 chest-lead minimum. In comparing the healthy subject and athlete, the body-surface potential distribution of the healthy subject first shows right ventricle excitation followed by left ventricle excitation, as shown in Fig. 5. Excitation results in local waveform positive peaking that is reflected in the potential distribution. Potential due to right ventricle excitation attains a positive peak and subsequently decreases to a negative peak after 40 ms. Meanwhile, left ventricle excitation is delayed relative to right ventricle excitation. The positive potential peak due to left ventricle excitation occurs 10 ms after the positive peak due to right ventricle excitation. Subsequently, right ventricle excitation produces a negative potential peak. Potential peaks of healthy subjects were verified to occur in the sequence: (1) right ventricle positive potential; (2) left ventricle positive potential; (3) right ventricle negative potential; and (4) left ventricle negative potential. Further, the potential of the R-wave peak was on the order of 3 mV for healthy subjects. As shown in Fig. 6, the athlete’s potential distribution showed left ventricle excitation following right ventricle excitation the same as for the healthy subject. However, at the time-point when positive potential due to left ventricle excitation reaches a maximum, the right ventricle simultaneously shows a peak in negative potential. Subsequently, a condition of residual negative potential for both the right and left ventricles is shown. Accordingly, for an athletic subject believed to have cardiopulmonary function superior to that of the healthy subject, potential peaks occurred in the sequence: (1) right ventricle positive potential; (2) right ventricle negative potential and left ventricle positive potential; and (3) left ventricle negative potential. Namely, a potential distribution pattern unique to the well-conditioned athlete was found that has simultaneous right ventricle negative peaking and left ventricle positive peaking. We attribute recognition of this previously unnoted result to the high temporal resolution of the present body-surface electrocardiograph system, and anticipate further clarification of the effects of cardiopulmonary endurance level on heart muscle as well as on the impulse conducting system.

As shown here, high-resolution data-acquisition and 3-D graphical representation simplifies body-surface potential distribution comparison with healthy subjects, and enables recognition of significant distinctions beyond individual differences. In particular, the tendency for positive peaking and negative peaking to occur simultaneously in well-conditioned athletes was made apparent. For a given subject, the time delay between potential peaking events can be easily judged by viewing the 3-D animated representation. The time delay between positive and negative peaks may be used, for example, as an indicator of cardiopulmonary capacity (or vitality). The difference between body-surface potential maxima and minima may be used to gage “heart age” in the same manner as previously suggested for establishing indicators of “heart age” using ultrasonic imaging (Masugata et al. 2007).

#### Body-surface potential pattern change with age

**Fig. 7:**
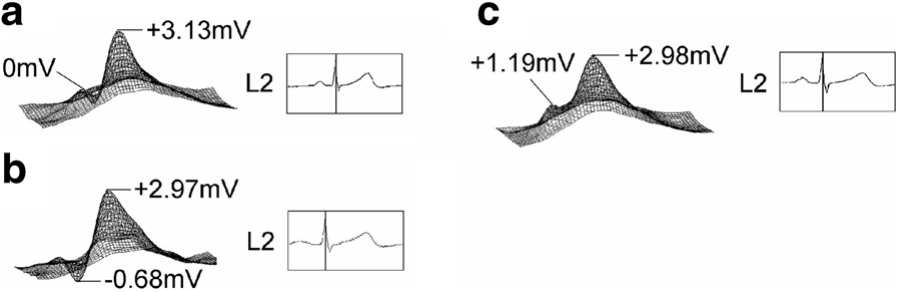
Body-surface potential for a single individual (male) at age 55 (**a**), 60 (**b**), and 65 (**c**) showing degradation of left ventricle positive peaking with age; standard ECG bipolar L2 (limb) lead waveforms are shown for reference

Next, change in the body-surface potential distribution patterns with age is examined. Body-surface potential for a single individual (Japanese male) was measured at three separate ages, which were age 55 shown in Fig. 7a, age 60 shown in Fig. 7b, and age 65 shown in Fig. 7c. It should be pointed out that this individual had no pre-existing conditions, and made no major vocational or life-style changes during the 10 year span of measurements. Figure 7 shows body-surface potential topography at the time of left ventricle positive peaking along with standard ECG bipolar L2 (limb) lead waveforms. While the L2 lead showed essentially the same waveform at each age point, comparison of left ventricle positive peak potential distributions showed peak potential reduction with age as well as evolution from a steep peak to a more gradual incline. This result supports the notion that positive cardiac electromotive force decreases with age. Although age-related changes have been investigated using the 12-lead ECG as well as earlier body-surface electrocardiograph systems (Mizuno 1966; Taccardi et al. 1998), normal-case adult age-distinctions have markedly gone unnoticed. We believe this is a result of the low spatial resolution of those systems. In contrast, reduction in peak potential with age and body-surface potential topography change from steep to gradual sloping can be intuitively grasped via the high resolution 3D animation of the present system. Accordingly, it is possible that age-related functional degradation of the heart due to the advancement of conditions such as hardening of the arteries may be verifiable in a dynamic manner.

#### Myocardial infarction warning signs

Although the onset of myocardial infarction has been divided into four stages, and each stage has been associated with QRS characteristics observable using the standard 12-lead ECG, it has been pointed out that the extent of the blocked regions may be underestimated (Flowers 1987; Sullivan et al. 1978). BSPM has also been applied to investigate myocardial infarction and the use of analytical representations such as Q maps (equipotential contour maps) has been suggested for diagnosis of myocardial infarction (Taccardi et al. 1998). Currently, those methods do not extend to complete identification of the region of necrosis (cell or tissue death). We note that acute myocardial infarction has been defined as sudden irreversible cellular dysfunction (necrosis) of cardiac muscle tissue due to pathologically advanced ischemia (diminished blood supply).

**Fig. 8:**
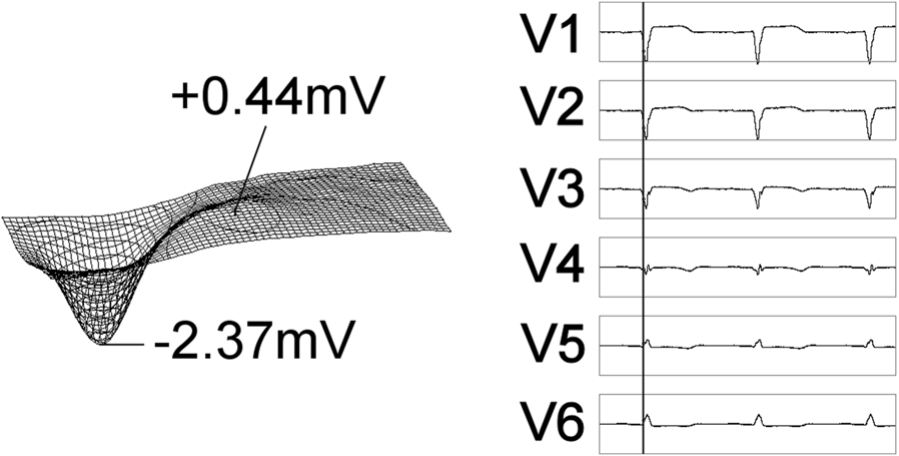
Body-surface potential for a subject with myocardial infarction and 80 % blockage of the coronary artery showing flattening (to two peaks) of the peak associated with left ventricle activity

Figure 8 shows graphical representation of body-surface potential at a given time-point for a subject with anterior myocardial infarction. This subject was diagnosed (by other methods) as having 80 % blockage of the left anterior descending coronary artery. The time-varying body-surface potential pattern of the present system showed negative potential only with slight positive potential developing at the point of maximum left ventricle excitation. The peak associated with left ventricle activity was flattened to a plateau with an elliptical outline (contour), and interestingly the topography of that region displayed a two maxima characteristic. Development of a region with no electrical activity (or conduction), which is a region of heart muscle necrosis, is a plausible explanation for this peak-flattening, two maxima phenomenon. In this case of anterior myocardial infarction, loss of precordial cardiac electromotive force was also confirmed. We believe the present system can perform a useful function in discovering regions of necrosis, and accordingly suggest that the system may be able to point out warning signs of myocardial infarction.

## Conclusions

Possible applications (e.g. development of indices to gauge the state of cardiopulmonary health) for a high spatial and temporal resolution body-surface electrocardiograph system have been surveyed. Although several decades have passed since the first appearance of research papers describing the principles of body-surface electrocardiography, its lack of acceptance is likely due to factors such as system size and complexity, challenging electrode attachment procedures, and the fact that useful applications are still generally unknown. The study reported here responds to those criticisms, and we present a high-resolution body-surface electrocardiograph system that is easier to use and capable of more accurate measurement and analysis than previously reported systems. Just as understanding of standard 12-lead ECG waveforms has evolved over time, general understanding of the formidable amount of information embedded in body-surface potential measurements is not expected immediately. However, accurately acquired potential distributions displayed dynamically in three dimensions (by the present system) organize the large data-sets into animated clips and should accelerate the learning process. The system measures data hidden by filtering, reveals phenomena undetectable by the 12-lead ECG, and overcomes a major barricade to practical application and general acceptance of body-surface electrocardiography, which is the troublesome, time consuming attachment of a large number of electrodes.

## Abbreviation

BSPM: body-surface potential mapping
